# Biomechanical analysis of different osteosyntheses and the combination with bone substitute in tibial head depression fractures

**DOI:** 10.1186/s12891-016-1118-4

**Published:** 2016-07-15

**Authors:** Martin C. Jordan, Christina Zimmermann, Sheridan A. Gho, Soenke P. Frey, Torsten Blunk, Rainer H. Meffert, Stefanie Hoelscher-Doht

**Affiliations:** Department of Trauma, Hand, Plastic and Reconstructive Surgery, University Clinics of Wuerzburg, Oberduerrbacher Strasse 6, 97080 Wuerzburg, Germany; Biomechanics Research Laboratory, School of Medicine, Faculty of Science, Medicine and Health, University of Wollongong, Northfields Avenue, Wollongong, NSW 2522 Australia; Department of Orthopaedics and Trauma Surgery, University Clinics of Bonn, Sigmund-Freud-Strasse 25, 53127 Bonn, Germany

**Keywords:** Tibial head fracture, Biomechanical test, Static test, Cyclic test, Tibial fracture fixation

## Abstract

**Background:**

Tibial head depression fractures demand a high level of fracture stabilization to prevent a secondary loss of reduction after surgery. Elderly individuals are at an increased risk of developing these fractures, and biomechanical investigations of the fractures are rare. Therefore, the aim of this study was to systematically analyze different types of osteosyntheses in combination with two commonly used bone substitutes.

**Methods:**

Lateral tibial head depression fractures were created in synthetic bones. After reduction, the fractures were stabilized with eight different treatment options of osteosynthesis alone or in combination with a bone substitute. Two screws, 4 screws and a lateral buttress plate were investigated. As a bone substitute, two common clinically used calcium phosphate cements, Norian® Drillable and ChronOS™ Inject, were applied. Displacement of the articular fracture fragment (mm) during cyclic loading, stiffness (N/mm) and maximum load (N) in Load-to-Failure tests were measured.

**Results:**

The three different osteosyntheses (Group 1: 2 screws, group 2: 4 screws, group 3: plate) alone revealed a significantly higher displacement compared to the control group (Group 7: ChronOS™ Inject only) (Group 1, 7 [*p* < 0.01]; group 2, 7 [*p* = 0.04]; group 3, 7 [*p* < 0.01]). However, the osteosyntheses in combination with bone substitute exhibited no differences in displacement compared to the control group. The buttress plate demonstrated a higher normalized maximum load than the 2 and 4 screw osteosynthesis. Comparing the two different bone substitutes to each other, ChronOS™ inject had a significantly higher stiffness and lower displacement than Norian® Drillable.

**Conclusions:**

The highest biomechanical stability under maximal loading was provided by a buttress plate osteosynthesis. A bone substitute, such as the biomechanically favorable ChronOS™ Inject, is essential to reduce the displacement under lower loading.

## Background

In orthopaedic surgery an increasing number of older patients are presenting for treatment of fractures, and this trend is likely to continue as the population ages. In this older patient group, tibial head fractures account for 10 % of all fractures [1]. Due to metaphyseal bone loss, depression fractures, especially of the lateral tibial plateau, frequently occur and need to be treated operatively [2, 3]. After reduction of the depressed articular fracture fragment, a metaphyseal bone defect remains. Filling the defect with an autologous iliac crest bone graft is not possible in elderly patients because of fatty degeneration of the iliac crest bone [4]. Instead, bone substitutes are used. Although the postoperative rehabilitation scheme typically includes a postoperative weight bearing of 15–20 kg after surgery of tibial head depression fractures, a secondary loss of reduction appears in up to 14 % of all cases under loading [5]. Furthermore, the risk of a posttraumatic arthritis of the knee increases if an intraarticular gap remains [6]. Therefore, it is imperative to ensure that the bone substitute used to fill a metaphyseal bone defect is highly stable.

When comparing commonly used calcium phosphate bone substitutes to a traditional bone graft, biomechanical studies have revealed an equal or even better primary stability for the bone substitutes [7, 8]. In previous biomechanical investigations of tibial head depression fractures, the need to use a bone substitute, preferably a drillable substitute, to reduce a secondary loss of reduction of the depressed articular fracture fragment has been demonstrated [9, 10]. Despite this, no studies, which have compared possible osteosyntheses and the effect of different bone substitutes in tibial head depression fractures, could be located. Rather, biomechanical studies analyzing the stability of osteosyntheses in other types of tibial head fractures are more readily available. For example, in split fractures of the lateral tibial plateau, a higher stability for 3 screws in the jail technique compared to conventional 2 screws has been shown [11]. In split depression tibial plateau fractures, advantages of plate designs with placement of subchondral screws to support the depressed articular fracture fragment compared to plates without subchondral screws has been verified, which could also offer a relevant treatment option to provide high stability for pure depression fractures of the tibial head [12]. Older patients often cannot follow the postoperative partial weight bearing of 15–20 kg, described above and a higher stability provided by the osteosynthesis would be desirable to reduce a secondary loss of reduction, especially of the depressed articular fracture fragment.

A systematic biomechanical evaluation of different options is needed to provide more information of the type of osteosynthesis and the effect of a combination with bone substitute for treating tibial head depression fractures. Therefore, the aim of this study was to systematically analyze different options of osteosyntheses alone and in combination with two commonly used bone substitutes for treating tibial head depression fractures. We hypothesized that, especially under maximal loading of the tibial plateau, a plate osteosynthesis alone provides a higher stability compared to screw osteosyntheses. Furthermore, we expected a two screw osteosynthesis demonstrating the lowest stability under maximal loading compared to a plate and to four screws. Corresponding to a previous biomechanical study, we hypothesized, that the bone substitute has no influence on the stability in each type of osteosynthesis under maximal loading, but is essential to reduce the loss of secondary reduction of the depressed articular fracture fragment despite the type of osteosynthesis [9]. We expected that there is no difference in our testing series between the two commonly used calcium phosphate cements (Norian® Drillable and ChronOS™ inject).

## Methods

### Fracture generation

**Fig. 1:**
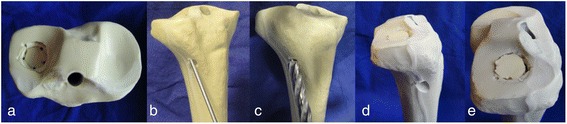
Lateral tibial plateau depression fracture and reduction. Lateral tibial plateau depression fractures (**a**) were generated in a fracture model. The fractures were reduced anatomically (**d**, **e**) by placement of a cannulated ram under the depressed articular fracture fragment (**b**, **c**)

Seventy two synthetic bones (Synbone 1110, SYNBONE®, Switzerland) were used as specimens because they have similar biomechanical qualities to elderly human bones as proved in a previous study [10, 13, 14]. The bones were cut at the diaphysis 20 cm below the tibial plateau and embedded in 5° valgus in a custom made device [15]. Lateral tibial head depression fractures were created by applying an axial load centrally on the lateral tibial plateau with an indentor after setting five predetermined breaking points in a 12-mm-diameter circle. The endpoint of fracture induction was set at a depth of 15 mm measured from the articular surface. All specimens were examined macroscopically and by taking x-rays. Only lateral tibial head depression fractures (type AO: 41-B2.2) were included in this study. The fractures were then reduced indirectly using the conventional arthroscopically supported reduction and internal fixation (ARIF), which is a commonly used operative technique for those fractures in our department [3]. The depressed fracture fragment was indirectly restored anatomically by K-wire guided cannulated ram (Fig. 1).

### Experimental groups

**Fig. 2:**
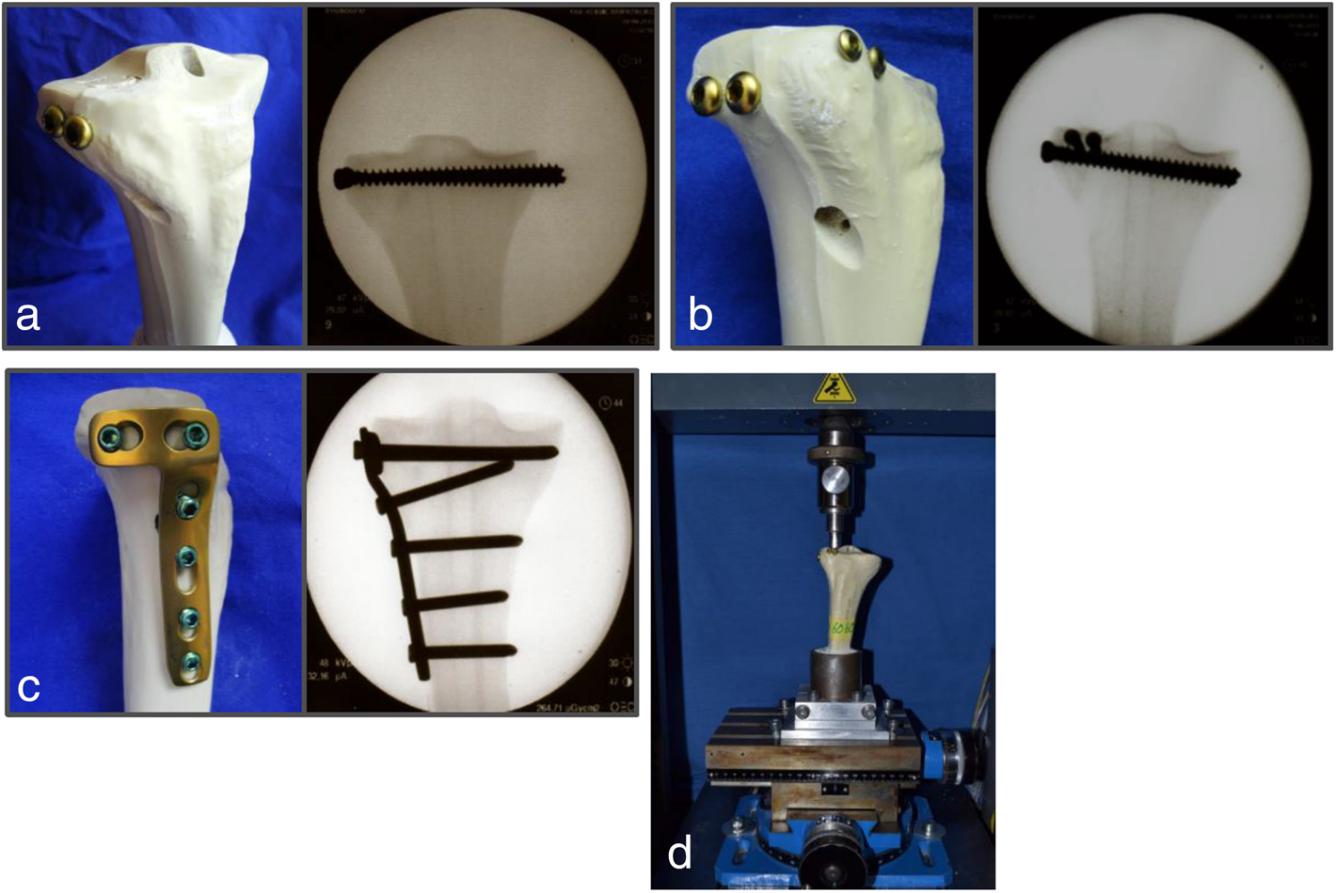
Types of osteosyntheses. The fractures were stabilized with three different osteosyntheses: **a** 2 screws, **b** 4 screws in the jail technique, and (**c**) lateral angle stable L-buttress plate. After stabilization an axial load was applied by an indentor on the lateral tibial plateau (**d**)

After reduction the specimens were randomly divided into eight different groups of stabilization (Table 1). Three osteosyntheses, 2 screws, 4 screws in the jail technique and a lateral angle stable L-buttress plate, were tested alone (Fig. 2) and in combination with two commonly used bone substitutes, ChronOS™ inject and Norian® Drillable (Synthes GmbH, Umkirch, Germany). ChronOS™ inject alone was applied as a bone substitute in a control group. Both bone substitutes are calcium phosphate cements: ChronOS™ inject is a ß-tricalcium phosphate bone substitute, whereas Norian® Drillable is a fiber reinforced calcium phosphate bone filler. After fracture stabilization, the specimens were examined using x-rays to assess the fracture reduction and the position of the osteosyntheses and the bone substitute. The same experienced orthopaedic surgeon performed all osteosyntheses. In each group, 9 specimens were tested.

**Table 1:** Experimental groups

Group	Osteosynthesis	Bone substitute
1	2 Screws	---
2	4 Screws	---
3	Buttress Plate	---
4	2 Screws	ChronOS
5	4 Screws	ChronOs
6	Buttress Plate	ChronOs
7	---	ChronOs
8	4 Screws	Norian

The eight different groups of fracture stabilization are shown

### Biomechanical test set-up

Physiologically, the tibial plateau is mainly affected by axial forces during normal gait [16, 17]. Therefore, an axial loading test set-up was deemed most appropriate for this experiment. After fracture stabilization, the specimens were placed in the material testing machine Zwick Roell Z020, Zwick GmbH & Co. KG, Ulm, Germany and an axial load was applied with an indentor on the lateral tibial plateau. The number of cycles and the loading conditions used were identified during a pre-testing series, in which 15 specimens with a 4 screw osteosynthesis over 10,000 cycles with different loading levels and with alternating increased loads were tested. The loading level for the cyclic testing phase like described in other biomechanical studies in tibial head fractures, also simulating typical postoperative partial weight bearing conditions with around 20 kg, also deemed to be reasonable for our tests.

After 10 settling cycles from 20 to 125 N, a load from 20 to 250 N was applied 3000 times at 25 mm/min [15, 18, 19]. Load-to-failure tests were conducted at the conclusion of the cyclic loading tests. The three primary outcome variables were displacement of the articular depression fracture fragment under cyclic loading (mm), the maximum load (N) and the stiffness under maximal loading (N/mm). The tests were performed in the material testing machine Zwick Roell Z020, and the data was recorded using a computer data recording system (software testXpert II, version 3.0, Zwick GmbH & Co. KG, Ulm, Germany). In the recorded load-displacement curve, maximum load was defined as the highest load measured during the Load-to-Failure tests and the stiffness as the ascending slope of the elastic deformation (linear slope at the beginning of the Load-to-Failure tests). The displacement, measured by the traverse of the material testing machine from the starting point (exactly on the articular depressed fracture fragment) was calculated at the loading peak (250 N) on settling cycle 10 and on measuring cycle 3000. The maximum load of the Load-to-Failure tests was also normalized to the maximum load of the native bone for fracture generation (calculated in percent of the maximum load of the native bone by the authors).

### Statistical analysis

The number of specimens for the experimental groups was estimated by power analysis using a significance level of 5 % and a power of 80 %. The calculation of effect size *d* was based on the results of a comparable pilot study. Descriptive statistics (means and standard deviations) for the three primary outcome variables were initially calculated for each of the eight experimental groups. Normal distribution of the data for each group was tested using the Shapiro-Wilk test. Normally distributed data were then evaluated using a one-way ANOVA design whereas data that were not normally distributed were analyzed using the Kruskal-Wallis test. The groups with two different bone substitutes (i.e. Group 5 and 8 in Table 1) were compared separately using an independent samples *t*-test after confirmation of normal distribution. The statistical analyses were conducted using IBM® SPSS® Statistics 21 with the level of significance set at *p* < 0.05.

## Results

The results of all groups are shown in Table 2.

**Table 2:** Results

Groups	Displacement measuring cycles [mm]	Displacement settling + measuring cycles [mm]	Maximum load [N]	Normalized maximum load [%]	Stiffness [N/mm]
1	1.4 ± 0.6	4.4 ± 1.4	1957 ± 348	148 ± 33	289 ± 51
2	1.1 ± 0.2	2.0 ± 0.6	3067 ± 257	231 ± 25	309 ± 61
3	3.7 ± 3.8	10.4 ± 2.8	3813 ± 556	337 ± 53	406 ± 99
4	1.1 ± 0.4	2.8 ± 0.8	2603 ± 317	203 ± 34	275 ± 44
5	0.8 ± 0.4	1.9 ± 1.2	3391 ± 281	262 ± 74	478 ± 149
6	0.7 ± 0.3	1.4 ± 0.9	3378 ± 443	289 ± 77	297 ± 114
7	0.5 ± 0.2	1.1 ± 0.6	2290 ± 325	196 ± 35	348 ± 101
8	1.0 ± 0.2	3.2 ± 0.9	3463 ± 444	247 ± 16	307 ± 62

The results (mean and standard deviation) of all groups are shown in Table 2

### Displacement

**Fig. 3:**
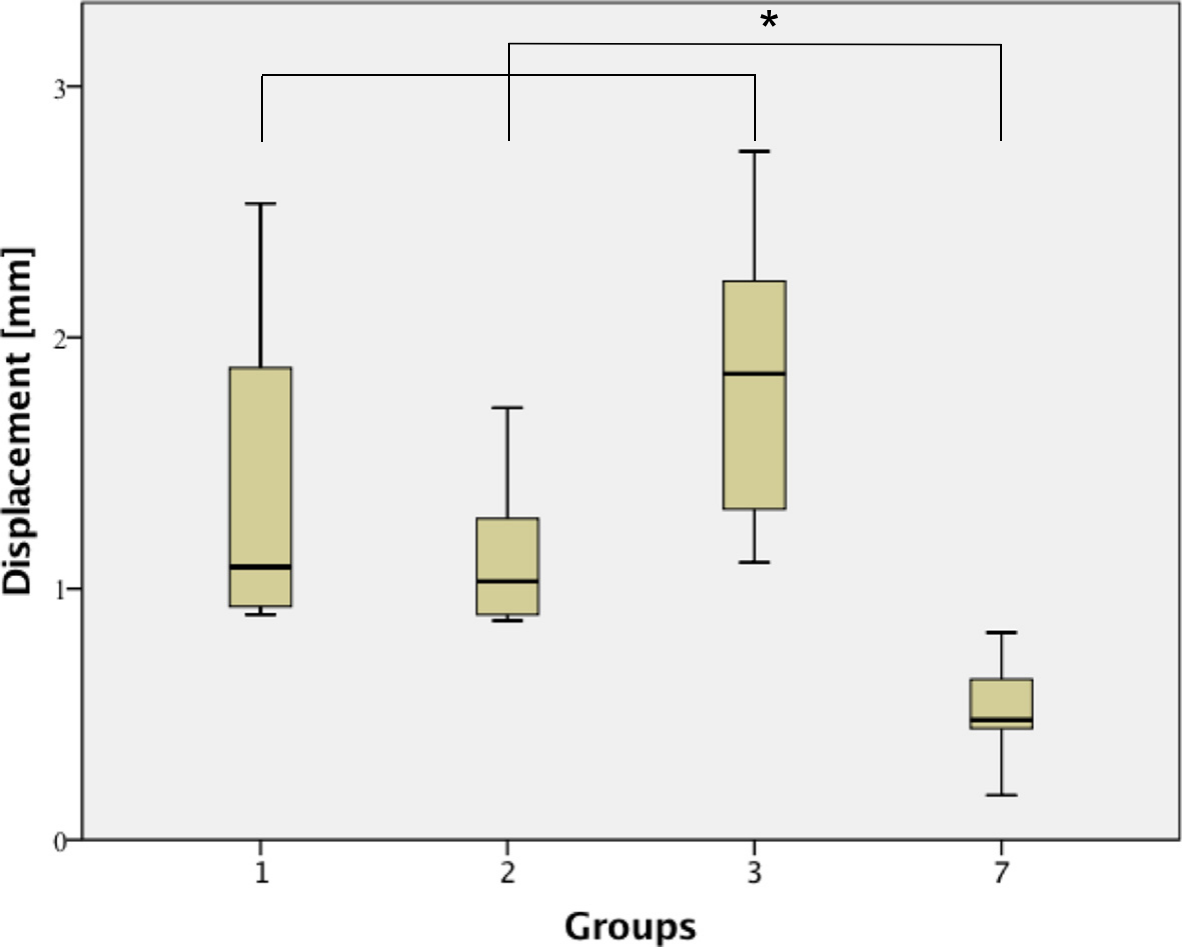
Displacement under cyclic loading. The osteosyntheses (Group 1–3) alone revealed a significantly higher displacement compared to the control group with bone substitute (ChronOS™ inject, group 7) alone (Group 1, 7 [*p* < 0.01]; group 2, 7 [*p* = 0.04]; group 3, 7 [*p* < 0.01]). An asterisk indicates a significant between-group difference (*p* < 0.05)

All groups stabilized with an osteosynthesis alone (Groups 1–3) displayed a significantly higher displacement compared to the control group with bone substitute ChronOS™ inject alone (Group 7) (Group 1, 7 [*p* < 0.01]; group 2, 7 [*p* = 0.04]; group 3, 7 [*p* < 0.01]) (Fig. 3). However, the addition of bone substitute (ChronOS™ inject) to the osteosyntheses (Groups 4–6), resulted in similar displacement values, with no significant differences between these groups when compared to the control group with ChronOS™ inject alone (Group 7) (Group 4, 7 [n.s.]; group 5, 7 [n.s.]; group 6, 7 [n.s.]).

The 2 screw osteosynthesis and the 2 screw osteosynthesis with bone substitute showed no significant differences in displacement (Group 1, 4 [n.s.]). Also, the 4 screw osteosynthesis in the jail technique demonstrated no significant differences with or without bone substitute (Group 2, 5 [n.s.]). Conversely, when comparing the two groups stabilized with the lateral angle stable L-buttress plate, the group with bone substitute revealed a significantly lower displacement than the group without bone substitute (Group 3, 6 [*p* < 0.01]). For the 3000 measuring cycles, no significant difference could be found when comparing the two different bone substitutes, ChronOS™ inject and Norian® Drillable, in combination with the 4 screws in the jail technique (Group 5, 8 [n.s.]). However, when taking the 10 settling cycles into account, a significant difference was detected (Group 5, 8 [*p* = 0.03]).

### Maximum Load

**Fig. 4:**
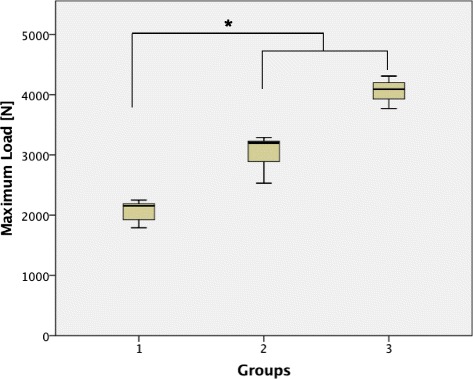
Maximum Load in Load-to-Failure tests. A significantly lower maximum load was determined for the 2 screw osteosynthesis (Group 1) compared to the 4 screw osteosynthesis (Group 2) and the lateral angle stable L-buttress plate (Group 3) (Group 1, 2 [*p* = 0.02]; group 1, 3 [*p* < 0.01]). An asterisk indicates a significant between-group difference (*p* < 0.05)

The lateral angle stable L-buttress plate without bone substitute and with bone substitute both demonstrated a significantly higher maximum load compared to the control Group 7 (Group 3, 7 [*p* < 0.01]; group 6, 7 [*p* < 0.01]). The 4 screws in the jail technique in combination with bone substitute revealed a significantly higher maximum load than the control group with ChronOS™ inject alone (Group 5, 7 [*p* < 0.01]). No significant differences were found between the groups with two different bone substitutes (Group 5, 8 [*p* = 0.7]). The 2 screw osteosynthesis demonstrated a lower maximum load compared to the 4 screws and lateral angle stable L-buttress plate (Group 1, 2 [*p* = 0.02]; group 1, 3 [*p* < 0.01]) (Fig. 4). The relationships between the three osteosyntheses did not vary with the addition of bone substitute (Group 4, 5 [*p* < 0.01]; group 4, 6 [*p* < 0.01]; group 5, 6 [n.s.]). In addition to the significant differences shown in Fig. 4, the normalized maximum load was significantly different between the 4 screws and the lateral angle stable L-buttress plate (Group 2, 3 [*p* < 0.01]).

### Stiffness

**Fig. 5:**
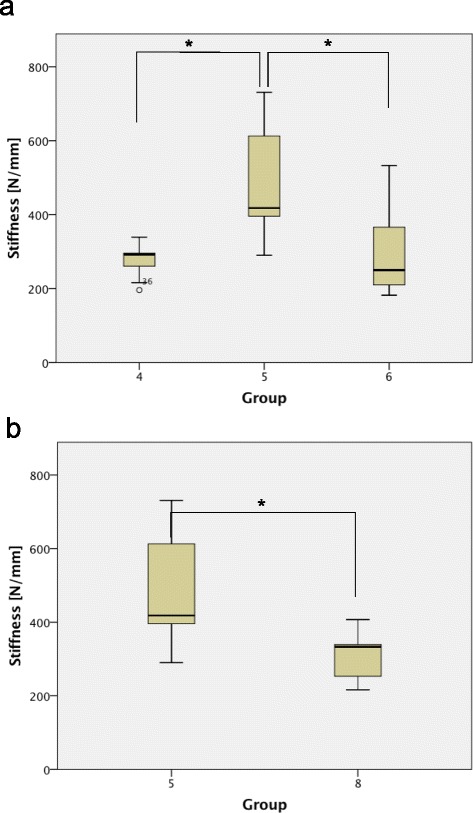
Stiffness in Load-to-Failure tests. Figure 5a: The 4 screws (Group 5) revealed a higher stiffness compared to the 2 screws (Group 4) and the plate osteosynthesis (Group 6), all in addition with ChronOS™ inject (Group 4, 5 [*p* < 0.01]; group 4, 6 [*p* < 0.01]). Fig. 5b: Comparing the two bone substitutes, the 4 screws with ChronOS™ inject (Group 5) demonstrated a higher stiffness than the 4 screws with Norian® Drillable (Group 8) (Group 5, 8 [*p* < 0.01]). An asterisk indicates a significant between-group difference

The jail technique with bone substitute exhibited significantly higher stiffness compared to the 2 screws with bone substitute (Group 4, 5 [*p* < 0.01]) and compared to the lateral angle stable L-buttress plate with bone substitute (Group 5, 6 [*p* < 0.01]) (Fig. 5a). No significant differences in stiffness were found when comparing Groups 1–6 with the control Group 7. The bone substitute ChronOS™ inject in combination with the jail technique revealed a significantly higher stiffness compared to the jail technique with Norian® Drillable (Group 5, 8 [*p* < 0.01]) (Fig. 5b).

## Discussion

Our biomechanical study is the first to provide systematic data to objectively compare primary stability of the osteosyntheses options in tibial head depression fractures, specifically 2 screws, 4 screws in the jail technique, and lateral angle stable L-buttress plate with subchondral screws. When comparing the three different osteosyntheses, the lateral angle stable L-buttress plate demonstrated the highest maximum load either with or without added bone substitute, which corresponded very well with our hypotheses. This indicates that for maximum load, the type of osteosynthesis is more crucial than the filling up of the metaphyseal bone defect with bone substitute. The plate and the 4 screw osteosynthesis provided a higher stability under maximal loading compared to only 2 screws.

Also well in agreement with our hypotheses, the use of bone substitute to fill up the bone defect is essential to reduce the displacement of the articular fracture fragment. Displacement results in the present study correspond well to previously published work [9]. Furthermore, results from the present study indicate that the completeness of filling up of the metaphyseal defect, especially directly under the articular fracture fragment, is affected by the type of osteosynthesis used. For 2 screws and 4 screws, no differences for the displacement were shown when comparing, respectively, the osteosynthesis with and without bone substitute. However, the plate osteosynthesis revealed a significant difference in displacement when comparing the combination with bone substitute to the plate osteosynthesis alone.

This study is the first to provide information about the biomechanical effect of the use of different bone substitutes in tibial head depression fractures. Two clinical commonly used bone substitutes were investigated in combination with 4 screws in the jail technique. Both were applied after screwing. Interestingly, in contrast to our hypothesis, for ChronOS™ inject, a lower displacement and higher stiffness was demonstrated relative to the Norian® Drillable. During testing, ChronOS™ inject exhibited a fixed contact with the synthetic bone spongiosa whereas Norian® Drillable was pressed out of the application channel. During maximal load testing of the Norian® Drillable group, the lateral tibial plateau stayed intact, whereas in the ChronOS™ inject group, the lateral tibial plateau broke completely away. These results indicate that the higher stability exhibited by the ChronOS™ inject may be provided at the expense of an undesirable mode of failure.

Tibial head depression fractures demand a high level of stability of the osteosynthesis, particularly in older patients, to prevent a secondary loss of reduction during postoperative loading. A remaining intraarticular gap also increases the risk of degenerative joint disease [6]. Well-established options for fracture stabilization are a 2 screw osteosynthesis and 4 screws in the jail technique [2, 3]. The screws are placed subchondral to support the reduced articular fracture fragment. In a biomechanical investigation, lateral angle stable L-buttress plates, which enable the placement of screws subchondral, exhibited a higher stability in split depression fractures of the tibial plateau [12]. In our hypotheses, we considered a plate osteosynthesis to provide also for tibial head depression fractures the highest stability of fracture fixation. Considering older patients often do not follow postoperative partial weight-bearing schemes, a high stability under maximal loading would be favorable. From a biomechanical point of view, the use of a lateral angle stable L-buttress plate would be the best treatment option in cases when high loading postoperatively is expected. The 4 screws in combination with bone substitute provided in our study the highest stiffness compared to the 2 screws and the plate and a high maximum load. However, the risk of wound healing problems by the more extensive soft tissue preparation required for a plate osteosynthesis must be taken into consideration on a case-by-case basis.

Filling up the defect with calcium phosphate cement instead of iliac crest bone graft in combination with a 2 screw osteosynthesis was shown already in a clinical trial as a good alternative to conventional plate osteosynthesis and iliac bone grafting [20]. The group with the bone substitute revealed in the study of *Keating* et al. a lower secondary loss of reduction in the long-term follow-up compared to the autologous bone graft [20]. This clinical study corresponds well to previous published biomechanical studies, which have revealed an equal or even better primary stability for the bone substitutes [7, 8].

Although it would be preferable to use real human bones, this was not feasible given the large number of specimens that were required for this systematic biomechanical analysis. The synthetic bone used for this study had a cortical and a trabecular structure like a human bone and exhibited similar values in the fracture simulation as human bones as shown in a previous study, in which different types of synthetic tibiae and human tibiae were biomechanically compared to each other related to the fracture model used in this study [10]. Furthermore, an advantage of using synthetic bones is a lower interspecimen variation. Anyway, the limitation of using an in vitro study to simulate physiological conditions is acknowledged. Nevertheless, this study contributes valuable biomechanical information about the primary stability of different treatment options of tibial head depression fractures under loading conditions simulating a partial weight bearing.

## Conclusions

Lateral tibial head fractures require a high level of stability of the osteosynthesis in combination with a bone substitute to fill up the remaining metaphyseal defect after reduction, particularly in older patients. This study provides valuable biomechanical information about three different possible osteosyntheses; 2 screws, 4 screws in the jail technique and lateral angle stable L-buttress plate, as well as information about the effect on the stability of two commonly used bone substitutes. The lateral angle stable L-buttress plate demonstrated a higher stability under maximal loading compared to the 2 and 4 screws, whereas the use of bone substitute was essential to reduce displacement of the depressed articular fracture fragment. Although biomechanically, ChronOS™ inject was favorable compared to Norian® Drillable, a total breaking of the lateral tibial plateau under maximal loading using ChronOS™ inject is not desirable, particularly in the event of a secondary operation. Based on the present results, conclusions can be drawn with respect to the necessary treatment required to ensure the bone substitute used to fill a metaphyseal bone defect is highly stable.

## Abbreviations

AO, Association of the Study of Internal Fixation; ARIF, arthroscopically supported reduction and internal fixation; n.s., no significance
